# Human Bocavirus Infection Markers in Peripheral Blood and Stool Samples of Children with Acute Gastroenteritis

**DOI:** 10.3390/v10110639

**Published:** 2018-11-15

**Authors:** Zaiga Nora-Krukle, Anda Vilmane, Man Xu, Santa Rasa, Inga Ziemele, Elina Silina, Maria Söderlund-Venermo, Dace Gardovska, Modra Murovska

**Affiliations:** 1 Institute of Microbiology and Virology, Rīga Stradiņš University, 5 Ratsupites St., LV-1067 Riga, Latvia; zaiga.nora@rsu.lv (Z.N.K.); anda.vilmane@rsu.lv (A.V.); santa.rasa@rsu.lv (S.R.); modra@latnet.lv (M.M.); 2 Department of Virology, University of Helsinki, 3 (PL21), 00014 Helsinki, Finland; man.xu@helsinki.fi (M.X.); maria.soderlund-venermo@helsinki.fi (M.S.-V.); 3 Children’s Clinical University Hospital, 45 Vienibas Avenue, LV-1004 Riga, Latvia; inga.ziemele@gmail.com (I.Z.); dace.gardovska@rsu.lv (D.G.); 4 Department of Paediatrics, Rīga Stradiņš University, 16 Dzirciema St., LV-1067 Riga, Latvia; sil.elina.23@gmail.com (E.S.)

**Keywords:** human bocavirus, children, acute gastroenteritis

## Abstract

Human bocaviruses (HBoVs) 1–4 belong to the Parvoviridae family, and they infect the respiratory or gastrointestinal tracts in children. We investigated the prevalence of HBoV1–4 DNAs in the blood and stool samples, and of HBoV1–4 IgG and IgM in the plasma samples, of children presenting with acute gastroenteritis (AGE). In addition, we identified HBoV co-infections with the five most frequent gastrointestinal pathogens. A total of 83 paired blood and stool samples were collected from children aged five years or less. Infection markers of HBoV1, 2, or 3 (viral DNA in blood and/or stool and/or antibodies) were detected in 61 out of 83 (73.5%) patients. HBoV1, 2, or 3 DNA as a monoinfection was revealed in 18.1%, 2.4%, and 1.2%, respectively, and 21.7% in total. In 56.1% of the HBoV DNA-positive patients, the presence in stool of another virus—most frequently norovirus or rotavirus—was observed. In conclusion, this study, for the first time, illustrates the prevalence and genetic diversity of HBoVs in Latvian children with gastroenteritis, and shows a widespread distribution of these viruses in the community. HBoV1 and 2 are commonly found as single infectious agents in children with AGE, suggesting that the viruses can be as pathogenic by themselves as other enteric agents are.

## 1. Introduction

Acute gastroenteritis (AGE) is common and affects all age groups around the world, and is the second-leading cause of child morbidity among pediatric infectious diseases. Globally, nearly 1.7 billion episodes of childhood diarrheal disease, and 525,000 deaths, occur annually in children less than five years of age [1].

Latvian Centre for Disease Prevention and Control data show that AGE is the fifth leading cause of morbidity overall, causing 3.7% of all illnesses in children during their first year of life. In children under one year of age, the incidence of AGE in the past five years (from 2012 to 2016) has increased, from 804 episodes in 2012 to 1059 episodes in 2016 [2].

Viruses such as rotavirus (RoV), noroviruses (HNoV), enteric adenoviruses (HAdV), and astroviruses (HAstV) are the major causes of AGE, but in many patients, despite various diagnostic methods, the etiological agent of the disease remains unidentified [3,4]. 

There are four HBoVs in the Parvoviridae family, and the Parvovirinae subfamily, belonging to the *Bocaparvovirus* genus, of which HBoV1 causes mild to severe respiratory tract infections mainly in children under the age of five. HBoV1 was first revealed in pooled nasopharyngeal aspirates of children in 2005, but can be found also in stool samples [5,6,7]. A few years later, in 2009, HBoV2 and HBoV3, and in 2010 also HBoV4, were discovered in stool samples of children [8,9,10]. HBoV2 is more frequently found in stool samples than in respiratory tract samples, but its association with AGE is disputed [8,9,11,12]. HBoV2 has only occasionally been found in the respiratory tract [13,14]. To date there is no confirmed association between HBoV3 or HBoV4 and AGE or respiratory tract infections [10,12,14,15].

The aim of this study was to investigate the detection rates of the respiratory and enteric HBoVs, as well as to determine co-infections with the five most frequent gastrointestinal pathogens in children presenting with gastroenteritis, in order to identify the possible role of HBoVs in the etiology of the disease in Latvia. This is the largest study of HBoVs in AGE where viral DNA findings in stool and blood samples have been compared with serology.

## 2. Materials and Methods

### 2.1. Patients and Samples

This study was performed in the Infectious Diseases Department of the Children’s Clinical University Hospital of Riga, Latvia from November 2013 to April 2017. Children who were hospitalized and who fulfilled the criteria for AGE (with diarrhea and/or vomiting, febrile temperature (i.e., axillary temperature higher or equal to 37.5 °C), with the duration of acute illness of less than 10 days) were included in this prospective study. The median duration of hospitalization was two days. On inspection, symptoms of acute respiratory tract infection (ARTI) in combination with AGE were also determined in some of the patients. Exclusion criteria were: Patients with chronic diseases and patients with bacterial gastroenteritis.

Altogether, 83 children were enrolled in the study. Of these 83 children, 51 (61.4%) were male and 32 (38.6%) were female (*p* = 0.0051). The median age was 19 months; 8 patients (9.6%) were less than 6 months old, 15 (18.1%) were 6–12 months old, 35 (43.2%) patients were 12–24 months old, and 25 (30.1%) were 2–5 years old. Pediatricians divided the patients into two groups: The AGE group (patients with gastrointestinal symptoms only) of 61 patients and the AGE–ARTI group (patients with symptoms of both AGE and ARTI) of 22 patients.

Stool and blood samples were obtained from all 83 children and studied for the presence of HBoVs and five gastro-intestinal viruses (HNoV genogroup I (HNoVGI) and II (HNoVGII), RoV, HAdV, HAstV, and sapovirus (HSaV)).

All procedures performed in the study involving human participants were in accordance with the ethical standards of the institutional and/or national research committee and with the 1964 Helsinki declaration and its later amendments or comparable ethical standards. The study protocol was approved by the Ethics Committee of the Rīga Stradiņš University (ethical approval issued on 30 May 2013; permission code: 25/30.05.2013). Written informed consent was received from all parents or guardians of participating children.

### 2.2. Nucleic Acid Extraction and PCR Analysis

DNA from whole blood was extracted using phenol-chloroform extraction, and from stool specimens using commercially available QIAamp DNA Stool Mini Kit (Qiagen, Valencia, CA, USA) according to the manufacturer’s protocol. The quantity of DNA was measured spectrophotometrically, and the quality of the DNA was proved using β-globin gene polymerase chain reaction (PCR). HBoVs were detected by nested PCR (nPCR), using primers amplifying the VP1/2 gene sequence of all four HBoVs, as described previously [10], followed by electrophoretic analysis of the PCR products in a 1.7% agarose gel. The positive qualitative nPCR results were verified and the HBoV loads were evaluated by a commercially available real-time quantitative PCR detection kit for HBoV genomes according to the manufacturer’s protocol (human bocavirus viral protein (VP) gene genesig standard kit, Genesig, Primerdesign Ltd., Southampton, UK). All stool samples were screened for five human viruses (HNoVGI and HNoVGII, RoV, HAdV, HAstV, HSaV) using a multiplex real-time PCR gastrointestinal virus assay (GI-Virus Assay, Seegene, Seoul, Korea).

### 2.3. Nucleotide Sequence Analysis

The HBoV-positive nPCR products were purified by MinElute PCR Purification Kit (Qiagen, Germany) and sequenced with ABI Prism 3100 Genetic Analyzer (Applied Biosystems, Foster City, CA, USA). Sequences were analyzed initially using program Chromas 2.5.1. and after that compared to the reference strains using the Basic Local Alignment Search Tool (BLAST).

### 2.4. VLP-Based Enzyme Immunoassay

HBoV1–4 IgG and IgM class antibodies were measured in all plasma samples from the children with AGE or AGE–ARTI, by in-house enzyme immunoassays (EIA) based on biotinylated virus-like particles (VLPs) of recombinant major capsid protein (VP3) at the Department of Virology, University of Helsinki, as described previously [16,17]. To verify that the IgG result was specific to each HBoV, a blocking reaction with VLPs of the heterologous HBoVs was performed in the plasma samples, for example, in HBoV1 IgG EIA, blocking in plasma was done with HBoV2 and HBoV3 non-biotinylated VLPs, and in HBoV2 IgG EIA, with HBoV1 and HBoV3 non-biotinylated VLPs [17,18]. The positivity cut-offs in competitive and non-competitive IgG EIA were 0.095 (mean +3SD) and 0.151 (mean +3SD), respectively, and in non-competitive IgM EIA, it was 0.131 (mean +3SD).

### 2.5. Statistical Methods

The statistical analysis was performed by GraphPad Prism 7.0 software, La Jolla, CA, USA (Fisher’s exact test, Mann–Whitney test, ANOVA). A *p* value < 0.05 was considered statistically significant.

## 3. Results

In total, HBoV-specific antibodies and/or DNA was detected in 61 out of the 83 (73.5%) patients included in the study. HBoV DNA in blood and/or stool samples was detected in 42 out of 83 (50.6%) patients, whereas nine out of 83 (10.8%) patients were IgM-positive and 51 out of 83 (61.4%) patients were IgG positive.

In the AGE group 33 out of 61 (54.1%) children were positive for HBoV DNA; 15 out of 33 (45.5%) children in blood samples only, seven out of 33 (21.2%) children in stool samples only, and 11 out of 33 (33.3%) children in both blood and stool samples. Sequencing results revealed that blood harbored only HBoV1 DNA, which was found in 26 patients; however, in stool samples HBoV1 DNA was detected in 11 patients, HBoV2 DNA in five patients, HBoV3 DNA in two patients, and HBoV4 DNA in none of the patients (Table 1).

In the AGE–ARTI group, of the nine out of 22 (40.9%) HBoV DNA-positive patients, HBoV DNA was detected in blood samples only in six out of nine (66.7%) patients, and in both blood and stool samples in three out of nine (33.3%) cases. None of the children in the AGE–ARTI group harbored viral DNA only in stool samples. Sequencing data affirmed that all detected virus-specific sequences, in both blood and stool samples, of the AGE–ARTI group belonged to HBoV1. There was no statistically significant difference between viral DNA findings in the two specimen types of the two groups (*p* > 0.05).

In the whole patient cohort, viral loads of ≥1 copies/µg DNA was detected in 15 (42.9%) out of 35 HBoV1 DNA-positive blood samples, with a median (IQR) of 58.69 (202.3–4.88) copies/µg DNA, and in 16 (72.7%) out of 22 HBoV DNA-positive stool samples, with a median (IQR) of 10.12 (144.7–3.95) copies/µg DNA.

### 3.1. HBoV-Specific Antibodies and DNA in Blood and Stool Samples

In the AGE group, HBoV-specific IgG antibodies were found in 36 out of 61 (59.0%) patients. Among them, 24 out of 36 (66.7%) patients were specific for HBoV1, of which two (5.6%) patients also had IgG against HBoV2 and two other patients against HBoV3. In turn, HBoV2-specific IgG antibodies alone were revealed in eight (22.2%) patients but none had only HBoV3 IgG. Five of the 24 IgG-positive patients (20.8%) also had HBoV1-specific IgM antibodies. None of the examined plasma samples in the AGE group was seropositive for HBoV4 (Table 1).

Ten (16.4%) out of the 61 AGE patients were HBoV-IgG-positive without having viral DNA in their blood or stool samples, whereas in seven out of 61 (11.5%) patients both HBoV IgG antibodies as well as DNA were detected in stool samples (Table 1). In four out of these seven patients, the IgG antibodies were specific to HBoV1. Two of these four patients were also IgG-positive for HBoV2 and one patient-for HBoV3. In 12 out of 61 (19.7%) patients, HBoV1 viremia as well as HBoV-specific IgG was found, but in nine patients the IgG was HBoV1-specific. Interestingly, two of these 12 patients had IgG against HBoV2 only and one against both HBoV1 and 2. Seven of the 61 (8.2%) patients were HBoV-seropositive and had HBoV DNA in both blood and stool samples. Five of those seven patients were IgG-positive for HBoV1 and two patients for HBoV2. Finally, in seven out of the 61 (11.5%) other patients, HBoV DNA in blood and/or stool samples occurred in seronegative individuals. Five children with HBoV1 DNA in blood and/or stool samples had HBoV1-specific IgG and IgM (Table 1).

In the AGE–ARTI group, nine (40.9%) out of 22 patients had HBoV-specific IgG antibodies, but no viremia or DNA in stool samples. In seven out of those nine patients, the IgG was specific to HBoV1, in one to HBoV2, and in one to both HBoV1 and HBoV2. Four out of 22 patients had both HBoV1 viremia and HBoV1-specific IgG antibodies, one of which was also seropositive for HBoV2. Three (13.6%) out of 22 patients had HBoV DNA both in blood and stool samples, and in two of them HBoV1- and 2-specific antibodies were also found (Table 1). Four patients from the AGE–ARTI group had IgM antibodies to either HBoV1 or HBoV2, whereas three patients were seronegative, but viremia had HBoV DNA in blood samples, or in both blood and stool samples.

In the whole study group, the seroprevalences of HBoV1, 2, and 3 were 50.6%, 16.9%, and 2.4%, respectively, the differences being statistically significant (*p* < 0.0001; *p* < 0.0001; *p* = 0.0027, respectively).

### 3.2. Co-Infections

All 83 stool samples were re-tested for five gastrointestinal viruses (HNoV GI and HNoVGII, RoV, HAdV, HAstV, and HSaV) by a multiplex real-time PCR. One or more of these enteric viruses were detected in 44 of the 83 patient stools; 33 out of 61 among the AGE children and 11 out of 22 among the AGE–ARTI children.

In the AGE group, 15 out of 43 (34.9%) patients had HBoV DNA and/or antibodies and also one more of analyzed enteric viruses, whereas eight (18.6%) patients had a genomic sequence of two additional enteric viruses (Table 2). The most frequently co-detected virus was HNoVGII, which was present in 16 out of 43 (37.2%) stool samples, followed by RoV in 12 out of 43 (27.9%), HAdV in two out of 43 (4.7%), and HAstV in one out of 43 (2.3%) stool samples. HNoVGII and RoV was detected significantly more frequently than HAdV or HAstV (*p* = 0.0003 and *p* < 0.0001, and *p* = 0.0068 and *p* = 0.0016, respectively) (Table 2). HBoV DNAs in stool samples were detected in 19 out of 61 (31.1%) patients, among whom HBoV monoinfection was revealed in eight patients, others had co-infection with one or two enteric viruses. In eight out of 61 (13.1%) patients of the AGE group, no viruses were detected in their stool samples.

In the AGE–ARTI group, HBoV DNA in stool samples was revealed in three out of 22 patients, from them one patient had HBoV monoinfection. Ten out of 18 (55.6%) patients had HBoV DNA and/or antibodies as well as one of five more analyzed enteric viruses genomic sequences, and none with two or more viruses. The co-presence of HBoV1 DNA with HNoVGII or RoV RNA was detected in eight and one stool sample(s), respectively (*p* = 0.0178). HNoVGII was revealed more often than HAdV, which was also found in one stool sample (*p* = 0.0178) (Table 2).

All 83 patients were divided into four age groups to analyze HBoV monoinfection and co-infections with tested enteric viruses (Table 3). Patients aged 0–6 months had HBoV monoinfections only, but patients in the age groups from 6–60 months had co-infection with HNoV GII significantly more often than with RoV (*p* = 0.0438). Co-infection with HAdV was significantly less common than with HNoVGII (*p* < 0.0001).

## 4. Discussion

This study was set up to elucidate the association of HBoVs with pediatric gastroenteritis and to determine the occurrence of co-infection with five of the most frequent acute gastroenteritis-causing viruses. The recruited patients were divided into two groups—children with AGE and children with AGE and ARTI. It is a common clinical experience that symptoms of both those clinical conditions may occur simultaneously.

The detection rate of HBoV infection markers in our study cohort was 73.5%. One of the explanations for such a high percentage is that our study reflects the results from both viral DNA and antibody findings, which were not always overlapping in each patient. However, a longitudinal study of weekly oral fluid samples from infants, analyzed by PCR for HBoV1 DNA, in America revealed that 76% of the children experienced a primary HBoV1 infection during the first 18 months of their life [19]. Another point is the inclusion criteria for patients participating in the study. Accordingly, we included only those patients in whom several bacterial and/or viral causative agents for AGE or AGE–ARTI were not identified in the clinic. The patients were thus pre-selected.

This is the first study so far where the methods of molecular biology and serology are combined, analyzing blood, plasma, and stool samples from all the recruited patients. In the AGE group, HBoV DNA was detected in blood or in stool samples, or in both simultaneously, but in the AGE–ARTI group there were no cases in which any HBoV was detected in stool samples only. The sequencing of the positive PCR products was used to identify the genotype of HBoV. Sequencing results were compared to the reference sequence from the National Center for Biotechnology Information database, revealing 99–100% similarity (Suppl. 1). In the current study, sequencing results showed that only the HBoV1 genomic sequence was found in the patients’ blood samples, though in stool samples HBoV2 and HBoV3 were also detected. Moreover, in the AGE–ARTI group, HBoV1 was also found in stool samples, demonstrating that HBoV1 is more a respiratory pathogen.

HBoV DNA in stool samples was detected in 25.3% of all patients, which is much higher than in other studies that have reported occurrence from 5.7% to 9.2% [12,20,21,22]. No statistically significant difference in frequency of HBoV1 DNA prevalence between the AGE and AGE–ARTI groups was found [i.e., 42.6% and 40.9%, respectively (*p* = 1)]. Conversely, HBoV2 or HBoV3 was detected only in seven out of 61 (11.5%) stool samples from the AGE patient group but none in the AGE–ARTI group (*p* = 0.1812). In a similar study from Finland, researchers analyzed nasal swab and stool samples from 955 children with ARTI, with both AGE–ARTI and AGE showing that HBoV2 and HBoV3, just like in our study, were more commonly found in stool samples [12].

In our study, a viral load higher or equal to one copy/µg DNA was detected in 42.9% of the HBoV-positive blood samples and in 76.2% of the positive stool samples. Results showed that a high viral load correlates with acuteness of the virus infection, because six out of nine HBoV1 and/or HBoV2 IgM-positive patients had elevated viral loads compared with HBoV IgM negative patients. There was no clinical difference between patients with high or low HBoV loads revealed in blood or stool samples. However, other studies, where respiratory samples were analyzed, showed correlations of high viral load with increasing disease duration or severity [7,23,24].

It has been shown that a combination of serological and PCR-based methodology is necessary for an accurate diagnosis of HBoV1 respiratory tract infection [16,25].

In 59.0% of patients from the AGE group and in 68.2% of patients from the AGE–ARTI group, HBoV-specific IgG antibodies were detected (*p* = 0.610). In the whole study cohort, the HBoV1 IgG seroprevalence is statistically higher than those of HBoV2 or HBoV3; in 50.6%, 16.9%, and 2.4%, respectively (*p* < 0.0001 for both). Additionally, the HBoV2 IgG prevalence is higher than that of HBoV3 IgG (*p* = 0.0027). HBoV4 DNA or specific antibodies were not found in this study, nor in our previous study [26], which allows us to conclude that HBoV4 is absent, or circulates rarely, in the Latvian child population.

Out of 21 patients with HBoV DNA-positive stool samples in the study cohort, five (23.8%) had an acute HBoV1 and two (9.5%) an acute HBoV2 infection, based on the presence of HBoV viremia and IgM, in addition to virus DNA, in stool samples.

There is much discussion among scientists and clinicians regarding whether HBoV1 co-infections may exacerbate the illness and have a meaningful role in the etiopathogenesis of the disease. To find out if there is a monoinfection of HBoV1–4, or if the patient is co-infected with other viruses, patients’ stool samples were examined for the presence of the most widespread causative viral agents of AGE: RoV, HNoV (GI and GII), enteric HAdV, HAstV, and HSaV. In our study cohort, HNoVGII was the most commonly detected virus in stool samples, followed by HBoV and RoV. These were detected in 34.9%, 25.3%, and 22.9% of the cohort, respectively. Taking into account only HBoV DNA findings in stool samples, HBoV as a monoinfection was revealed in 10.8% of 83 patients, whereas another virus as a co-infection was detected in 15.7% of the patients from the cohort. The number of co-infecting viruses in HBoV-positive cases ranged from one to two.

Results of the study show that most of the HBoV2-positive cases were patients with AGE without symptoms of respiratory tract infections. Also, HBoV2 positive cases were most frequently monoinfections, which was statistically significant (*p* = 0.0365), demonstrating its nature of being predominantly an enteric virus. Similar results have been reported by other research groups [19,27]. Previous studies have mostly detected HBoV1 in respiratory specimens and HBoV2 in stool samples [11,12,13]. However, HBoV1 is also often detected in stool samples and in some regions HBoV1 is the most frequently detected HBoV in stool samples [27]. This is consistent with the results of our study. It has been shown that HBoV1 first causes respiratory tract infection and then persists without any symptoms for several months, and a hypothesis is that thereafter the virus would infect the gastrointestinal tract, perhaps causing symptoms of gastroenteritis [28]. Alternatively, HBoV1 can be swallowed during acute respiratory disease and then appear in stools; however, it is known that diarrhea in small children might be an additional symptom in HBoV1 respiratory infection [12,29].

The present study, for the first time, illustrates the prevalence and genetic diversity of HBoVs in the Latvian child population with gastroenteritis, and demonstrates the widespread distribution of these viruses in the community. HBoV1 is commonly found as a single infectious agent in children with AGE, suggesting that the virus can be as pathogenic by itself as other enteric agents are. This study revealed that, in the AGE group, HBoV1 is the most common bocavirus detected in stools, leaving open the question regarding HBoV1’s ability to survive in the passage through the gastrointestinal tract, or acting as a causal agent of the disease.

## Figures and Tables

**Table 1:** Human bocavirus PCR and EIA results of children with acute gastroenteritis, or acute gastroenteritis with acute respiratory tract infection.

PCR Findings in Blood/Stool	*n*	IgM+			IgG+				IgM−/IgG−
		HBoV1	HBoV2	HBoV1 & HBoV2	HBoV1	HBoV2	HBoV1 & HBoV2	HBoV1 & HBoV3	
AGE group, *n* = 61									
HBoV1+/HBoV1+	6	2 ^a^			3				3
HBoV1+/HBoV2+	4	1 ^a^			1	2			1
HBoV1+/HBoV3+	1				1				
HBoV1+/HBoVs−	15	2 ^a^			9	2	1		3
HBoVs−/HBoV1+	5				4	1			
HBoVs−/HBoV2+	1					1			
HBoVs−/HBoV3+	1							1	
HBoVs−/HBoVs−	28				6	2	1	1	
AGE–ARTI group, *n* = 22									
HBoV1+/HBoV1+	3	1 ^a^		1 ^a^	1		1		1
HBoV1+/HBoV2+	0								
HBoV1+/HBoV3+	0								
HBoV1+/HBoVs−	6	1 ^a^	1 ^a^		3		1		2
HBoVs−/HBoV1+	0								
HBoVs−/HBoV2+	0								
HBoVs−/HBoV3+	0								
HBoVs−/HBoVs−	13				7	1	1		

+/− presence/absence of viral DNA in corresponding sample; AGE—acute gastroenteritis; ARTI—acute respiratory tract infection; PCR—polymerase chain reaction; IgG—immunoglobulin G [average absorbances ± standard deviation (range): HBoV1 1.560 ± 1.153 (0.107–3.950); HBoV2 0.460 ± 0.449 (0.134–1.794); HBoV3 0.852 ± 0.676 (0.162–1.65)]; IgM—immunoglobulin M [average absorbances ± standard deviation (range): HBoV1 0.582 ± 0.417 (0.151–1.382); HBoV2 0.146 and 0.185]; HBoV—human bocavirus; HBoV1 and HBoV2—antibodies against HBoV1 and 2 simultaneously; ^a^ patients with also the presence of IgG; *n*—number of patients.

**Table 2:** Presence of human bocaviruses and other enteric pathogens.

Virus Patient Groups	*n*	HAdV	HNoVGII	RoV	HAdV/HNoVGII	HNoVGII/RoV	HNoVGII/HAstV	WCI
AGE, HBoVs+	43	1	8	6	1	6	1	20
AGE, HBoVs−	18	1	3	5		1		8
AGE–ARTI, HBoVs+	18	1	8	1				8
AGE–ARTI, HBoVs−	4		1					3

+/− presence/absence of HBoV in corresponding sample; AGE—acute gastroenteritis; ARTI—acute respiratory tract infection; HAdV—adenovirus; HAstV—astrovirus; HBoV—human bocavirus; HNoVGII—norovirus genogroup II; RoV—rotavirus; WCI—without co-infection; *n*—number of patients.

**Table 3:** Presence of human bocaviruses and other enteric pathogens in patients in different age groups.

Viruses Age (Months)	*n*	HNoVGII	RoV	HAdV	HAstV
0–6 (HBoV+)	5				
0–6 (HBoV−)	3	1			
6–12 (HBoV+)	10	5	1	1	
6–12 (HBoV−)	5		1		
12–24 (HBoV+)	22	8	5		1
12–24 (HBoV−)	13	4	5	1	
24–60 (HBoV+)	24	11	7	2	
24–60 (HBoV−)	1				

+/− presence/absence of HBoV in corresponding sample; HBoV–human bocavirus; HNoVGII—norovirus genogroup II; RoV—rotavirus; HAdV—adenovirus; HAstV—astrovirus; *n*—number of patients. There was no statistically significant difference between the HBoV viral load in blood samples in the case of single and co-infections (*p* = 0.2721).

## Supplementary Materials

The following are available online at http://www.mdpi.com/1999-4915/10/11/639/s1, Supplement 1: Sequencing results.
