# Supplementary material for: Human Bocavirus Infection Markers in Peripheral Blood and Stool Samples of Children with Acute Gastroenteritis
Source: Viruses. 2018 Nov 15;10(11):639. doi: 10.3390/v10110639 (PMC6265904; doi:10.3390/v10110639)
Supplement: Supplementary file 1 [file viruses-10-00639-s001.pdf]

Supplement 1

A

Primary sequence No 1:
TTTGTANTCAATAAAGTGCAAAAAAACAAAAAAAATGAACCTAAACCAG
GAACCTCAAAAATGTCTGACACTGACATTCAAGACCAACAACCTGATACT
GTGGACGCACCACAAAACACCTCAGGGGGAGGAACAGGAAGTATTGGAG
GAGGAAAAGGATCTGGTGTGGGGATTTCCACTGGAGGGTGGGTCGGAGGT
TCTCACTTTTCANACAAATATGTGGTTACTAAAAACACAAGACAATTTATA
ACCACAATTCANAATGGTCACCTCTACAAAACANAGGCCATTGAAACAAC
AAACCAAAGTGGAAAATCACAGCGCTGCGTCACAACTCCATGGACATACT
TTAACTTTAATCAATACAGCTGTCACTTCTCACCACAGGATTGGCAGCGCC
TTACAAATGAATATAAGCGCTTCAAACCTAAAGCAATGCAAGTAAAGATT
TACAACTTGCAAATAAAACAAATACTTTCAAATGGTGCTGACACAAC

Reference sequence: Human bocavirus 1 isolate CBJ001 capsid protein VP1 and capsid protein VP2 genes, partial cds; Sequence ID: gi|727099172|KM378039.1; Length: 522; Number of Matches: 1; Identity: 99%.

Primary sequence No 2:
TTTTNTTTGCTACTCAATAAAGGTGCAAAAAAAACAAAAAAAAGTGAACC
TAAACCAGGAACCNNAAAAATGTCTGACACTGACATTCAAGACCNACNN
CCTGATACTGTGGACGCACCACAAAACACCTCAGGGGGAGGAACAGGAA
GTATTGGAGGAGGAAAAGGATCTGGTGTGGGGATTTCCACTGGAGGGTGG
GTCGGAGGTTCTCACTTTTCANACAAATATGTGGTTACTAAAAACACAAG
ACAATTTATAACCACAATTCANAATGGTCACCTCTACAAAACAGAGGCCA
TTGAAACAACAAACCAAAGTGGAAAATCACAGCGCTGCGTCACAACTCCA
TGGACATACTTTAACTTTAATCAATACAGCTGTCACTTCTCAC

Reference sequence: Human bocavirus 1 isolate V1445149 VP1 gene, complete cds; Sequence ID: gi|1443028968|MG680946.1; Length: 2016; Number of Matches: 1; Identity: 98%

B

Primary sequence No 3:
TAACGAGCCTAAACCAGGCACTTCAAAAATGTCTGAAAATGAAATCCAAG
ACCAACAACCATCTGACTCAATGGAAGAGCGAGGAGGAGGAGGAGGTGC
GACCGGTAGTGTGGGAGGGGGGAAAGGTTCTGGTGTGGGTATATCCACAG
GTGGCTGGGTAGGAGGCAGCTACTTCACTGACTCATATGTCATAACAAAA
AACACCAGACAATTTCTGGTAAAAATACAAAATGACCACAAATACAGAA
CTGAAAATATTATTCCAAGCAATGCTGGAGGAAAATCACAAAGATGCGTC
AGCACACCGTGGTCATATTTCAACTTCAATCAATACAGCAGTCATTTTTCA
CCACAAGACTGGCAGCGCCTAACAAATGAATATAAGCGCTTTAAACCTAG
AAAAATGCATGTAAAAATTTACAATCTACAAATAAAACAAATACTTTCAA
ATGGTGCTGACACTACA

Reference sequence: Human bocavirus isolate 9506 VP1 gene, partial cds; Sequence ID: gi|379069807|JN990592.1; Length: 529; Number of Matches: 1; Identity: 100%

Primary sequence No 4:
CTTTTTACTTTGCAACTCAATAAAGGTGCTAAAAAACCAAAAAATAACGA
GCCTAAACCAGGCACTTCAAAAATGTCTGAAAATGAAATCCAAGACCAAC
AACCATCTGACTCAATGGAAGAGCGAGGAGGAGGAGGAGGTGCGACCGG
TAGTGTGGGAGGGGGGAAAGGTTCTGGTGTGGGTATATCCACAGGTGGCT
GGGTAGGAGGCAGCTACTTCACTGACTCATATGTCATAACAAAAAACACC
AGACAATTTCTGGTAAAAATACAAAATGACCACAAATACAGAACTGAAA
ATATTATTCCAAGCAATGCTGGAGGAAAATCACAAAGATGCGTCAGCACA
CCGTGGTCATATTTCAACTTCAATCAATACAGCAGTCATTTTTCACCACAA
GACTGGCAGCGCCTAACAAATGAATATAAGCGCTTTAAACCTAGAAAAAT
GCATGTAAAAATTTACAATCTGCAAATAAAACAAATACTTTCAAATGGTG
CTGACACTACATACAACAAC

Reference sequence: Human bocavirus 2 strain W153, complete genome; Sequence ID: gi|158714105|EU082213.1; Length: 5204; Number of Matches: 1; Identity: 99%

C

Primary sequence No 5:
TTTTGCAACTCAATAAAGGTGCTAAAAAATCAAAAAACAACGAACCTAAA
CCAAGCACCTCAAAAATGTCTGAAAATGAAATTCAAGACCAACAGCCATC
AGAACCTAATGATGGCCAACGAGGAGGGGGAGGAGGTGCGACCGGCAGT
GTGGGAGGGGGGAAAGGTTCTGGTGTGGGTATATCCACAGGTAGATGGGT
AGGAGGCAGCTACTTTACTGACTCCTATGTCATAACAAAAAACACCAGAC
AATTTCTGGTTAAAATCCAAAACAACCATCAATATAAAACTGAAAATATA
ATTCCTTCCAATGGAGGAGGAAAATCACAAAGATATGTCAGCACACCATG
GTCATACTTTAACTTTAATCAATACAGCAGTCATTTCTCACCACAGGACTG
GCAGCGCCTAACAAATGAATACAAAAGATTCAGACCTAAAGGTATGCATG
TTAAAATCTACAATTTACAAATAAAACAGATTTTATCAAATGGTGCTGATG
TTACATACAACAACGATT

Reference sequence: Human bocavirus 3 strain HBoV-3/BRA/TO-57/Brazil/2014 isolate BRA/TO-57, complete genome; Sequence ID: gi|1352422202|MG953832.1; Length: 5155; Number of Matches: 1; Identity: 99%

Primary sequence No 6:
GACTTTTATTTTGCAACTCAATAAAGGTGCTAAAAAATCAAAAAACAACG
AACCTAAACCAAGCACCTCAAAAATGTCTGAAAATGAAATTCAAGACCAA
CAGCCATCAGAACCTAATGATGGCCAACGAGGAGGGGGAGGAGGTGCGA
CCGGCAGTGTAGGAGGGGGGAAAGGTTCTGGTGTGGGTATATCCACAGGT
GGATGGGTAGGAGGCAGCTACTTTACTGACTCCTATGTAATAACAAAAAA
CACCAGACAATTTCTGGTTAAAATCCAAAACAACCATCAATATAAAACTG
AAAGTATAATTCCTTCCAATGGAGGAGGAAAATCACAAAGATGTGTCAGC
ACACCATGGTCATACTTTAACTTTAATCAATACAGCAGTCATTTCTCACCA
CAGGACTGGCAGCGCCTAACAAATGAATACAAAAGATTCAGACCTAAAG

GTATGCATGTTAAAATCTACAATTTACAAATAAAACAGATTTTATCAAATG
GTGCTGATGTTACATACAACAA

Reference sequence: Human bocavirus strain HBoV3, complete genome; Sequence ID: gi|340746301|JN086998.1; Length: 5319; Number of Matches: 1; Identity: 99%

Supplementary material 1: Sequencing results: (A) Primary nucleotide sequences of human bocavirus 1 isolated from stool and blood samples of patients with gastroenteritis and corresponding reference strains; (B) Primary nucleotide sequences of human bocavirus 2 isolated from stool samples of patients with gastroenteritis and corresponding reference strains; (C) Primary nuceotide sequences of human bocavirus 3 isolated from stool samples of patients with gastroenteritis and corresponding reference strains.
